# Atorvastatin reduces lipopolysaccharide-induced expression of cyclooxygenase-2 in human pulmonary epithelial cells

**DOI:** 10.1186/1465-9921-6-27

**Published:** 2005-04-03

**Authors:** ShangJie Wu, Shu Duan, ShuiPing Zhao, Ying Cai, Ping Chen, Xiang Fang

**Affiliations:** 1 Division of Cardiovascular Disease, Department of Internal Medicine, The Second Xiangya Hospital, Center Southern University, Changsha, Hunan, China; 2 Division of Respiratory Disease, Department of Internal Medicine, The Second Xiangya Hospital, Center Southern University, Changsha, Hunan, China; 3 Department of Biochemistry, University of Iowa Carver College of Medicine, Iowa City, IA 52242, USA

**Keywords:** Cyclooxygenase-2, Lipopolysaccharide, Atorvastatin, Prostaglandin E_2_, Human pulmonary epithelial cell

## Abstract

**Objective:**

To explore the effects of atorvastatin on expression of cyclooxygenase-2 (COX-2) in human pulmonary epithelial cells (A549).

**Methods:**

A549 cells were incubated in DMEM medium containing lipopolysaccharide (LPS) in the presence or absence of atorvastatin. After incubation, the medium was collected and the amount of prostaglandin E_2 _(PGE_2_) was measured by enzyme-linked immunosorbent assay (ELISA). The cells were harvested, and COX-2 mRNA and protein were analyzed by RT-PCR and western-blot respectively.

**Results:**

LPS increased the expression of COX-2 mRNA and production of PGE_2 _in a dose- and time-dependent manner in A549. Induction of COX-2 mRNA and protein by LPS were inhibited by atorvastatin in a dose-dependent manner. Atorvastatin also significantly decreased LPS-induced production of PGE_2_. There was a positive correlation between reduced of COX-2 mRNA and decreased of PGE_2 _(r = 0.947, P < 0.05).

**Conclusion:**

Atorvastatin down-regulates LPS-induced expression of the COX-2 and consequently inhibits production of PGE_2 _in cultured A549 cells.

## 1. Introduction

Human pulmonary epithelial cell is one of major sources of productive inflammatory biomediators, such as prostaglandin E_2 _(PGE_2_), interleukin-6 (IL-6), in respiratory inflammatory diseases [1]. Cyclooxygenase-2 (COX-2) is an inducible enzyme that is expressed in response to inflammatory cytokines, and it is responsible for the synthesis of pro-inflammatory PGs such as PGE_2_. Increased expression of COX-2 and production of PGE2 have been found in pulmonary inflammatory disorders [2].

Statins is a class of compounds that decreases cholesterol synthesis via inhibition of 3-hydroxy-3methylglutaryl coenzyme A (HMG-CoA) reductase. Recently, anti-inflammatory effects of statins have been described [3]. For example, Atorvastatin reduces expression of the COX-2 in cultured vascular smooth muscle cells [4]. However, it is not clear whether Atorvastatin also affects COX-2 expression in human pulmonary epithelial cells. Because of importance of COX-2 in inflammatory respiratory diseases, we tested the effects of Atorvastatin on lipopolysaccharide (LPS)-induced expression of COX-2 in cultured human pulmonary epithelial cells.

## 2. Methods

### 2.1 Materials

Human pulmonary epithelial cell line (A549) was purchased from American Type Culture Collection (ATCC). Medium DMEM, trypsin, fetal bovine serum (FBS) and LPS were purchased from Sigma-Aldrich. ECL chemiluminescence reagents, COX-2 polyclonal antibody were purchased from Cayman Chemical Co. Anti-rabbit IgG, horseradish peroxidase linked whole antibody was obtained from Amersham LIFE SCIENCE. HECAMEG was from Vegatec (Villejuif, France). Trizol and electrophoresis reagents were from Promag Co. Atorvastatin was a gift from Beijing Honghui Medicine Co.

### 2.2 Cell culture

A549 cells were grown in DEME medium supplemented with 5% FBS, 100 u/ml penicillin, 100 u/ml streptomycin, and 50 μg/L amphotericin B. Cells were sub-cultured into six-well plates and maintained until sub-confluence. The medium was then replaced by a serum-free culture medium for 24 h prior to the addition of LPS and/or other reagents. The cells were then incubated with various concentrations of LPS for 9 h, or 10 μg LPS for different times. For atorvastatin experiments, the cells were incubated in the serum-free medium containing 10 μg LPS in the presence or absence of different concentrations of atorvastatin for 9 h.

### 2.3 PGE_2 _assay

After incubation, the medium was collected for measurement of PGE_2_. PGE_2 _was determined by enzyme-linked immunosorbent assays (ELISA, Shanghai Sun Biomedical Co. CV <10%).

### 2.4 RNA extraction, reverse transcription-polymerase chain reaction (RT-PCR)

COX-2 mRNA was measured by RT-PCR as previously described [5]. Briefly, total RNA from different experimental conditions was obtained by Trizol method (Life technologies) and the concentration of RNA was determined by an absorbance at 260 nm. For RT-PCR, 100 ng of RNA from different experimental conditions was applied to the access RT-PCR System. The following primers were used for COX-2: forward: 5'-AAG CTG GGA AGC CTT CTC TA-3' and reverse: 5'-TTT CCA TCC TTG AAA AGG CGC-3', which yielded products of 342 bp (50 sec at 55°C for annealing of the primers, 35 cycles), and CYCLOPHILIN A: forward: 5'-ATG GTC AAC CCC ACC GTG TTC TTC G-3' and reverse: 5'-CGT GTG AAG TCA CCA CCC TGA CAC A-3', which yielded products of 206 bp (50 sec at 55°C for annealing primers, 38 cycles). The DNA products from RT-PCR reactions were analyzed on a 4% polyacrylamide-urea gel in the same buffer. The polyacrylamide gels were dried and scanned using the ImageQuant densitometer (Gel Doc 2000, BioRad Co).

### 2.5 Western Blot analysis

After incubation, A549 cells were washed twice in phosphate-buffered saline, lysed in 200 μl lysis buffer (20 mM Tris/HCL, pH 7.5, 20 mM HECAMEG, 1 mM benzamidine). Protein content was determined by a microbicinchoninic acid assay (Pierce) with bovine serum albumin as standard. Western blot analysis was performed as described previously [6]. Briefly, the protein was separated by electrophoresis on a 10% polyacrylamide gel at 180 V for 45 min. After transfer to nitrocellulose, the membrane was blocked, incubated with a specific rabbit polyclonal antibody against COX-2 (1:1000). The blots were then incubated with a horseradish peroxidase-conjugated donkey anti-rabbit antibody (1:5000). Antibody labeling was detected by enhanced chemiluminescence. The films were scanned using an Arcus II Agfa scanner, and densitometric analysis was performed using Sigma Gel software.

### 2.6 Statistical analysis

Statistical analysis was performed with SPSS analysis (SPSS10.0 Software). PGE_2 _and RT-PCR data are presented as mean ± S.D., and the differences between the multiple treatment groups were analyzed by the one-way ANOVA, LSD test. Data were correlated by nonparametric Spearman's rank method. Probability values of 0.05 or less were considered to be statistically significant.

## 3. Results

### 3.1 Dose- and time-dependent effects of LPS on COX-2 mRNA expression and PGE2 production

**Figure 1:**
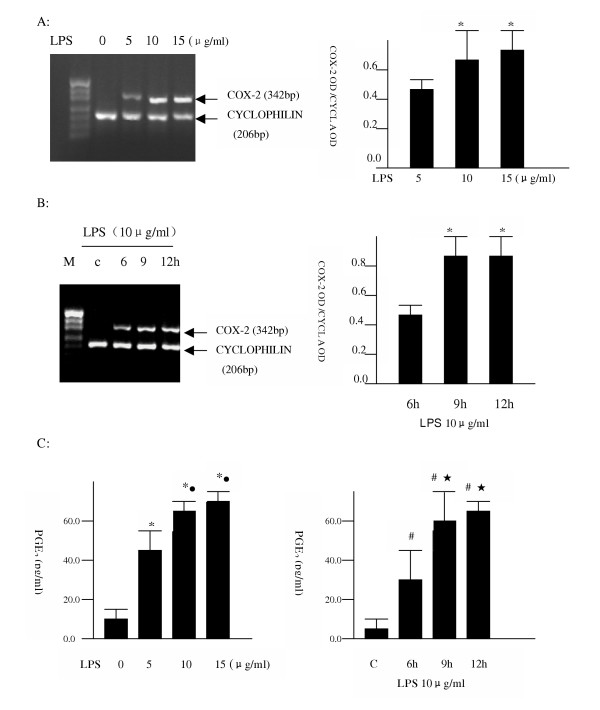
**A: **Dose-dependent effect of LPS on COX-2 mRNA expression. A549 cells were incubated with various concentrations of LPS for 9 h (top, * P < 0.05 vs LPS 5 μg/ml). **B: **Time-dependent effect of LPS on COX-2 mRNA expression. A549 cells were incubated with LPS (10 μg/ml) for various times (middle, * P < 0.05 vs 6 hrs group;). **C: **Time- and dose-dependent effects of LPS on PGE_2 _production (bottom, * P < 0.05 vs non-LPS group; ^• ^P < 0.05 vs 5 μg/ml LPS group; # P < 0.01 vs non-LPS group;  P < 0.05 vs 6 h group). These data were representative of three separate experiments.

To determine the concentration dependent effect of LPS, A549 cells were incubated with various concentrations of LPS for 9 h. RT-PCR analysis indicated that LPS increased the expression of COX-2 mRNA in a concentration-dependent manner (Figure 1A, **top**). Concentrations as low as 5 μg/ml LPS were effective in inducing expression of the COX-2 mRNA. To determine the time-dependent effect, A549 cells were incubated with LPS (10 μg/ml) for different times. The expression of COX-2 mRNA was increased by LPS as early as 6 h, the earliest time point tested. It reached a maximum induction by 9 h of incubation, and remained stable for at least 12 h, the longest time tested (Figure 1B, **middle**). LPS also increased the production of PGE_2_, a major cyclooxygenase product in A549 cells, in a time- and dose-dependent manner (Figure 1C, **bottom**). LPS (5 μg/ml) caused a 3-fold increase in amount of PGE_2_, and increased PGE_2 _was also observed as early as 6 h of incubation.

### 3.2 Effect of atorvastatin on LPS-induced expression of COX-2 mRNA and protein

**Figure 2:**
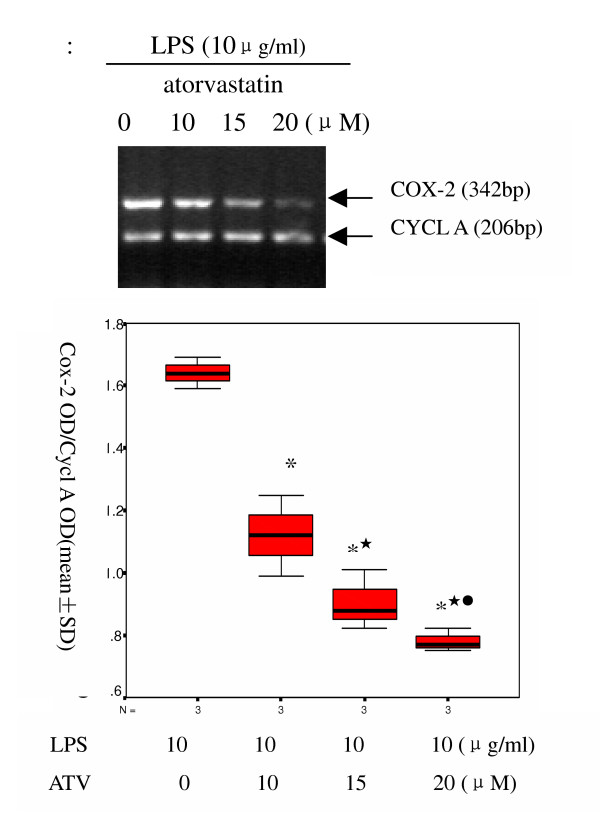
Effect of atorvastatin on LPS-induced expression of COX-2 mRNA. A549 cells were treated with various concentrations of atorvastatin in the presence of LPS (10 μg/ml) for 9 h. After incubation, total RNA were extracted and assayed by RT-PCR. A representative gel. (top) and relative density of gel. (bottom) are shown. * P <0.05 vs non- atorvastatin.  P < 0.05 10 μM vs atorvastatin group; ^• ^P < 0.05 vs 15 μM atorvastatin group.

**Figure 3:**
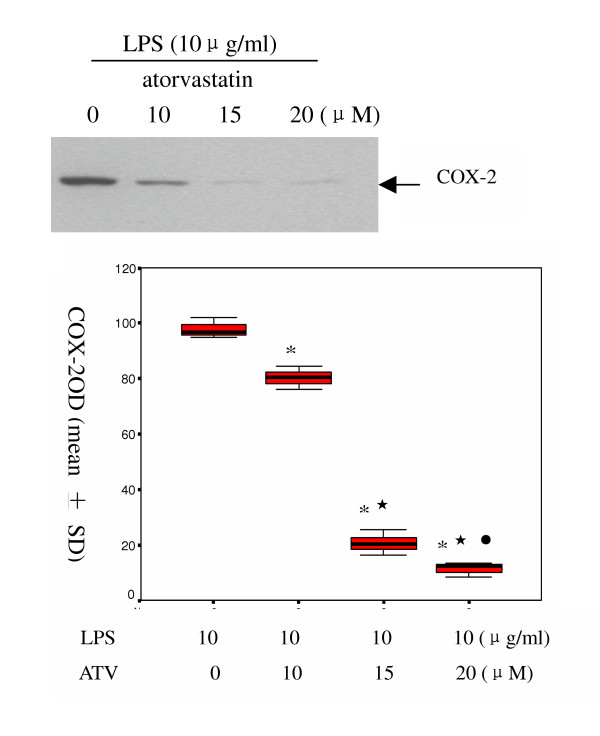
Effect of atorvastatin on LPS-induced expression of COX-2 protein. A549 cells were treated as described in Figure 2. After incubation, the cells were harvested, and sonicated. COX-2 protein in the cell lysates was detected by Western-blot using a specific antibody against COX-2. A representative western-blot gel. (top) and the density of COX-2 band (bottom) are shown. * P = 0.001 vs non atorvastatin;  P < 0.05 vs 10 μM atorvastatin; ^• ^P < 0.05 vs 15 μM atorvastatin group.

To determine whether atorvastatin affect the LPS-induced expression of COX-2, the cells were incubated with various concentrations of atorvastatin for 9 h in the presence of 10 μg/ml LPS. RT-PCR analysis indicated that LPS-induced expression of the COX-2 mRNA was decreased significantly by atorvastatin (Figure 2). Consistent with this observation, LPS-induced expression of the COX-2 protein was also inhibited by atorvastatin (Figure 3). Atorvastatin inhibited LPS-induced expression of COX-2 mRNA and protein in a dose-dependent manner.

### 3.3 Effect of atorvastatin on LPD-induced PGE_2 _production

Because atorvastatin decreased the expression of COX-2 mRNA and protein, we determined whether it also blocks PGE_2 _production. A549 cells were incubated with various concentrations of atovastatin in the presence of 10 μg/ml LPS for 9 hrs. After incubation, the medium was collected, and the amount of PGE_2 _in the medium was detected by ELISA. As shown in Table 1, atorvastatin decreased LPS-induced PGE_2 _production in a dose-dependent manner.

**Table 1:** The effects of atorvastatin on amount of PGE_2 _production

	LPS (10 μg/ml)			
Atorvastatin	0	10 μM	15 μM	20 μM
PGE_2 _(pg/ml)	58.3 ± 2.8	46.1 ± 3.0*	31.2 ± 0.7*	31.7 ± 3.6*

* P < 0.05 *vs *non-atorvastatin.

### 3.4 Correlations

According to Spearman's non-parametric rank correlation method, data analysis revealed that atorvastatin-mediated reduction of LPS-induced expression of COX-2 is correlated with a decrease in PGE_2 _production (r = 0.947, P < 0.05).

### 3.5 Time-dependent effects of atorvastatin on LPS-induced COX-2 expression and PGE2 production

**Figure 4:**
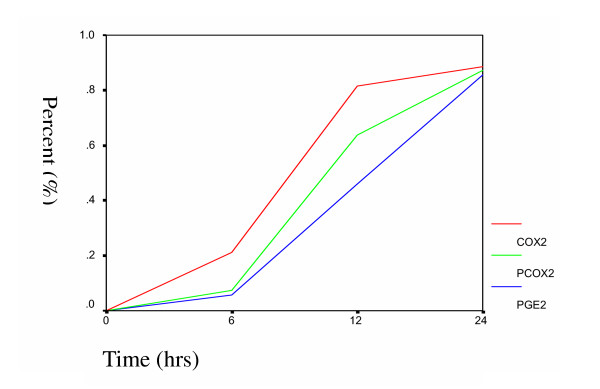
Time-dependent effects of atorvastatin on LPS-induced expression of COX-2 mRNA (red line), COX-2 protein (green line) and PGE_2 _production (blue line). The A549 cells were incubated with 10 μM atorvastatin in the presence of LPS (10 μg/ml) for different times. After incubation, COX-2 mRNA and protein were analyzed by RT-PCR and western-blot respectively, and the amount of PGE2 in the medium was determined by ELISA. Percent inhibitions by atorvastatin are shown.

We further investigated the time-dependent effect of atorvastatin on expression of COX-2 and PGE_2 _production. A549 cells were incubated with 10 μM atorvastatin in the presence of 10 μg/ml LPS for various times. Atorvastatin decreased the expression of COX-2 mRNA, protein, and PGE_2 _in a time-dependent manner (Figure 4). The result showed the similar time-dependent patterns in atorvastatin-mediated reduction of COX-2 mRNA, protein and PGE_2_, further suggesting a relationship between COX-2 expression and PGE_2 _production.

## 4. Discussion

Inflammatory cytokines as well as prostaglandins (PGs) play important roles in inflammatory process of respiratory system [7]. PGs are synthesized from arachidonic acid by a reaction catalyzed by cyclooxygenase. Two isoforms of this enzyme have been identified [8]. COX-1 is expressed constitutively in almost all tissues [9], and COX-2 is an inducible enzyme that is expressed in response to inflammatory cytokines [10]. Increased expression of COX-2 has been reported in human pulmonary epithelial cells under experimental inflammatory conditions [11]. In the present study, we also found that LPS induces expression of COX-2 mRNA and PGE2 formation in a dose- and time-dependent manner in A549 cells. These results suggested that expression of the COX-2 could be induced in A549 cells. Because the COX-2 is responsible for the synthesis of pro-inflammatory PGs such as PGE_2 _[10,11], an increased expression of COX-2 might play an important role in respiratory inflammatory processes.

HMG-CoA reductase inhibitors, which decrease the synthesis of cholesterol, have been shown to decrease the incidence of acute coronary events [12]. Recent studies suggest that the beneficial effects of statins on clinical events may be not related to its' effect on cholesterol synthesis. Statins affect endothelial cells, smooth muscle cells, monocyte- macrophage, vasomotor function, inflammatory responses, and plaque stability [13,14]. Anti-inflammatory action of statins might be related to the reduction of the production of pro-inflammatory cytokines. Statins inhibit the Ang II-induced secretion of interleukin-6 (IL-6) in cultured human vascular smooth muscle cells, and decrease production of IL-6, interleukin-1β in human umbilical vein endothelial cells [15]. Atorvaststin also down-regulates expression of COX-2 mRNA both *in vivo *and *in vitro *[4]. In this study, we found that atorvastatin significantly reduced LPS-induced expression of COX-2 mRNA in cultured A549 cells. Atorvastatin also significantly reduced LPS-induced PGE_2 _production. The correlation analysis indicated that there is a positive correlation between reduced expression of COX-2 and decreased PGE_2_. Furthermore, the patterns showing effects of atorvastatin on LPS-induced expression of COX-2 mRNA, protein and PGE_2 _production in different times were similar. These suggest that decreased production of PGE_2 _by atorvastatin is caused by down-regulation of COX-2 expression. In contrast to our observations, other study [16] showed that mevastatin and lovastatin increase expression of COX-2 and subsequent prostacyclin formation in human aortic smooth muscle cells. It appears that the effects of statins on expression of COX-2 might depend on the cell types or different statins used.

In conclusion, atorvastatin down-regulates LPS-induced expression of COX-2 and production of PGE_2 _in cultured A549 cells. These results suggest that HMG-CoA reductase inhibitors might have beneficial effects against respiratory inflammation.
